# Chemoprevention of malaria with long-acting oral and injectable drugs: an updated target product profile

**DOI:** 10.1186/s12936-024-05128-1

**Published:** 2024-10-18

**Authors:** Myriam El Gaaloul, Andre Marie Tchouatieu, Kassoum Kayentao, Brice Campo, Benedicte Buffet, Hanu Ramachandruni, Jean Louis Ndiaye, Timothy N. C. Wells, Celine Audibert, Jane Achan, Cristina Donini, Hellen C. Barsosio, Halidou Tinto

**Affiliations:** 1 MMV Medicines for Malaria Venture, Route de Pré-Bois 20, Post Box 1826, 1215 Geneva 15, Switzerland; 2 Malaria Research and Training Center, University of Sciences, Techniques and Technologies of Bamako, Bamako, Mali; 3 University Iba Der Thiam of Thiès, Thiès, Senegal; 4 Malaria Consortium, London, UK; 5 Kenya Medical Research Institute, Centre for Global Health Research, Kisumu, Kenya; 6 Department of Clinical Sciences, Liverpool School of Tropical Medicine, Liverpool, UK; 7 Clinical Research Unit of Nanoro, Institut de Recherche en Sciences de la Santé, Nanoro, Burkina Faso

**Keywords:** Malaria, Chemoprevention, Drug development

## Abstract

Malaria is preventable, but the burden of disease remains high with over 249 million cases and 608,000 deaths reported in 2022. Historically, the most important protective interventions have been vector control and chemopreventive medicines with over 50 million children receiving seasonal malaria chemoprevention in the year 2023. Two vaccines are approved and starting to be deployed, bringing additional protection for children up to 36 months. However, the impact of these currently available tools is somewhat limited on various fronts. Vaccines exhibit partial efficacy, are relatively costly, and not accessible in all settings. The challenges encountered with chemoprevention are barriers to acceptability and feasibility, including frequency of dosing, and the lack of options in the first trimester of pregnancy and for women living with HIV. Also, the emergence of resistance against chemopreventive medicines is concerning. To address these limitations, a target product profile (TPP) is proposed as a road map to guide innovation and to boost the quest for novel chemopreventive alternatives. This TPP describes the ideal product attributes, while acknowledging potential trade-offs that may be needed. Critically, it considers the target populations most at risk; primarily infants, children, and pregnant women. Malaria control and elimination requires appropriate chemoprevention, not only in areas of high endemicity and transmission, but also in lower transmission areas where immunity is declining, as well as for travellers from areas where malaria has been eliminated. New medicines should show acceptable safety and tolerability, with high and long protective efficacy. Formulations and costs need to support operational adherence, access, and effectiveness. Next generation long-acting oral and injectable drugs are likely to constitute the backbone of malaria prevention. Therefore, the perspectives of front-line experts in malaria prevention, researchers, and those involved in drug development are captured in the TPP. This inclusive approach aims at concentrating efforts and aligning responses across the community to develop new and transformative medicines.

## Background

Malaria is a preventable disease that nonetheless continues to cause significant global morbidity, mortality and economic hardship [1]. Indeed, 249 million cases and 608,000 deaths were reported in 2022, with over 94% of deaths occurring in sub-Saharan Africa [1]. Current malaria prevention measures encompass vector control, chemoprevention, and lately vaccination, but all these approaches have their limitations [2]. Advances across all interventions are required to adequately protect the entire population, prevent severe malaria and mortality, and progress towards elimination [3].

**Fig. 1:**
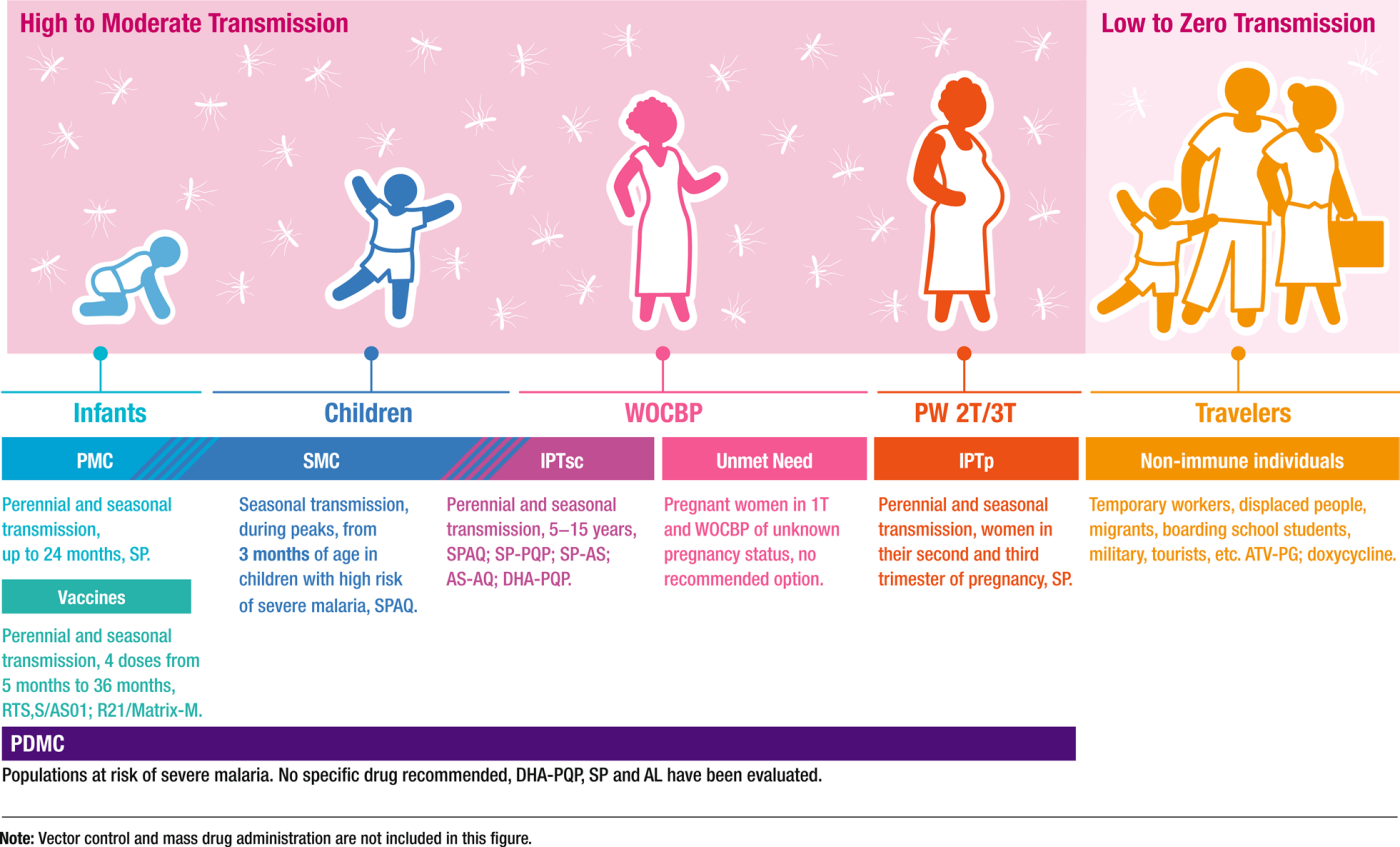
Overview of current malaria prevention interventions. *AL* artemether-lumefantrine, *AQ* amodiaquine, *AS* artesunate, *ATV* atovaquone, *DHA* dihydroartemisinin, *IPTp* intermittent preventive treatment in pregnancy, *IPTsc* intermittent preventive treatment in school-aged children, *PDMC* post-discharge malaria chemoprevention, *PG* proguanil, *PMC* perennial malaria chemoprevention, *PQP* piperaquine, *PW* pregnant women, *SMC* seasonal malaria chemoprevention, *SP* sulfadoxine-pyrimethamine, *T* trimester, *WOCBP* women of childbearing potential

Malaria prevention is targeted at populations most at risk of severe malaria living in moderate-to-high transmission areas (Fig. 1). The World Health Organization (WHO) recommends chemoprevention in children up to 15 years of age, including perennial malaria chemoprevention (PMC; previously intermittent preventive treatment of malaria in infants) in children up to 24 months of age, seasonal malaria chemoprevention (SMC) in children from 3 months of age, intermittent preventive treatment of malaria in school-aged children (IPTsc) aged 5–15 years, and post-discharge malaria chemoprevention following hospital treatment of children with severe malaria [2]. Additionally, intermittent preventive treatment of malaria in pregnancy (IPTp) is recommended in the second and third trimester [2]. PMC, IPTsc, and IPTp are reliant on sulfadoxine-pyrimethamine (SP) and SMC uses the combination of SP and amodiaquine (SPAQ). However, the effectiveness of these interventions is at risk from the potential spread of high-level SP resistance [4–7]. In addition, the operational complexity of a monthly 3-day dosing schedule in seasonal areas is significant, clearly pointing to the need for more simplified therapies. For travellers, atovaquone-proguanil is used for chemoprophylaxis but is not widely used within Africa.

Recently, two malaria vaccines, RTS,S/AS01 and R21/Matrix-M, have been approved for use by the WHO to protect infants from severe malaria [2, 8, 9]. Both vaccines require four doses for two years of coverage and are an important advancement [10–12]. However, they are limited by the need for a cold chain, the requirement for administration by trained personnel and potential vaccine hesitancy [13–17]. They are also relatively expensive, at a current price of almost $40 for RTS,S/AS01 and $16 for R21/Matrix-M for a four-dose course. Additionally, the evidence for RTS,S/AS01 indicates that protective efficacy varies by age and location [18], and is short lived [18, 19]. However, recent studies have shown that combining seasonal malaria vaccination with SMC yielded better results than either intervention given alone [20, 21]. Understanding how and where this combination can be effectively implemented at scale will be an important research question for the future.

The progress in protecting pregnant women has been inadequate [22]. Neither of the current malaria vaccines is approved for use in adults. Chemoprevention with SP is contraindicated in pregnant women living with HIV and in all women in the first trimester of pregnancy. Even for the second and third trimesters, the coverage of at least three SP doses during pregnancy is estimated as 42% globally but is often considerably lower [1]. Considering that annually over 35 million pregnancies occur in moderate to high transmission areas [1], this low coverage of IPTp and the lack of means to protect against infection during pregnancy represent a significant gap in therapeutics which needs urgent attention.

In areas where transmission is declining, populations will develop semi-immune status more slowly, moving the age distribution of disease up, with an increasing number of children over 5 years old at higher risk of severe disease [23, 24]. Consequently, SMC among 5–10-year-old children is being implemented in some countries [25, 26]. Climate change, either through displacement of people to new regions or because of changes in vector distribution and malaria seasonality may also cause non-immune populations to become threatened by malaria [1, 27–30].

In such a context, there is an urgent need for convenient, well-tolerated and efficacious alternative drugs addressing the limitations of current interventions and meeting the needs of a broader target population. A revised target product profile (TPP) outlining both the minimal acceptable and ideal profiles of chemopreventive drug combinations is proposed. The TPP aims to support go/no-go decisions through the development pathway, and describes target product labelling, thereby facilitating communication with regulators. This TPP was developed in an inclusive manner with field-based clinical experts, researchers, and drug developers. The overarching objective of the TPP is to propose a road map to guide and boost the development of a future generation of chemopreventive options.

### Current limitations of chemoprevention drugs

Chemoprevention currently uses a full therapeutic course of anti-malarial medicine at pre-scheduled times, irrespective of infection status, and covers a range of populations [2] (Fig. 1). The limitations of the different interventions can be considered together as they all address individuals at risk of malaria, with unknown infection status, and with the aim of clearing current and preventing future infections. However, there are specific key gaps in provision and critical unmet needs for each group.

The most widely adopted chemopreventive strategy in Africa is SMC. Although PMC and IPTsc have been piloted in several countries, widespread implementation has not followed. Barriers to implementation include practical issues such as the need to crush tablets, and poor integration with childhood health services [31, 32]. In contrast, SMC was administered to over 53 million children in 2023. This success relied on extensive training of community healthcare workers and community-based education to promote acceptability among parents [31, 32]. The intervention is highly cost-effective, reducing infection by fourfold in clinical trial settings in children under 5 years of age [33, 34]. However, where SMC has been deployed, shifts in the disease burden to older age groups have been detected [25, 26, 35, 36]. Consequently, MMV supports the expansion of SMC to children aged 5–10 years, as currently being implemented in some countries [25, 26]. For many years, SMC was not deployed in the seasonal zones of Eastern Africa, though this is now being tested [37–39]. However, within the malaria community, there is continued concern about the high prevalence of SP resistance molecular markers in some regions [40, 41]. There is also the risk that widespread deployment of SMC will drive further resistance selection to SP or to amodiaquine [41–43]. Another key limitation is the need to administer amodiaquine for three days per month [44, 45].

The adoption of chemoprevention in pregnancy has been limited. IPTp with at least three courses of SP is recommended after week 13 of pregnancy to protect women and reduce adverse outcomes [2, 46]. Despite the extremely low cost of IPTp and the single dose monthly schedule, adherence and coverage remains poor. The barriers to effective IPTp include gaps between antenatal care providers and malaria services, limited health promotion, and low acceptance both by pregnant women and healthcare providers [47–50]. This is partially explained by concerns about the anti-malarial efficacy of SP [51, 52]. Insufficient information about the benefits of IPTp and the importance of adhering to the recommended dosing schedule, traditional beliefs or preferences, and stigma or discrimination when seeking healthcare services also deter access to IPTp with SP [51]. The recently launched community IPTp guidelines address the missed opportunities for increasing IPTp coverage by promoting a community-based delivery approach [53]. Another important limitation is the lack of options for women living with HIV and the unmet need for all women in their first trimester of pregnancy. In the early stages of pregnancy, many women are hesitant to discuss their status or may not yet be aware of being pregnant, therefore all women of childbearing age with unknown pregnancy status should be ethically treated as potentially in the first trimester.

In summary, an ideal next generation oral medicine would have no cross-resistance to existing anti-malarial drugs, be administered as a single dose per month, to increase adherence and facilitate roll out and would be indicated for all populations at risk.

To address the need for infrequent dosing, the concept of long-acting injectables with small molecules also requires exploration [54]. For example, in pre-exposure prophylaxis (PrEP) for HIV, daily doses of cabotegravir have been replaced with an extended-release injectable suspension maintaining protection over 2 months [55]. Additionally, monoclonal antibodies (mAbs) currently in development for malaria show encouraging findings [56–58]. However, these are likely to provide protection against *Plasmodium falciparum* only, face challenges like vaccines in terms of a limited target population and cold chain deployment, and significant work is needed to bring the costs to an affordable level [59].

### Rationale for revising the target product profile (TPP)

**Fig. 2:**
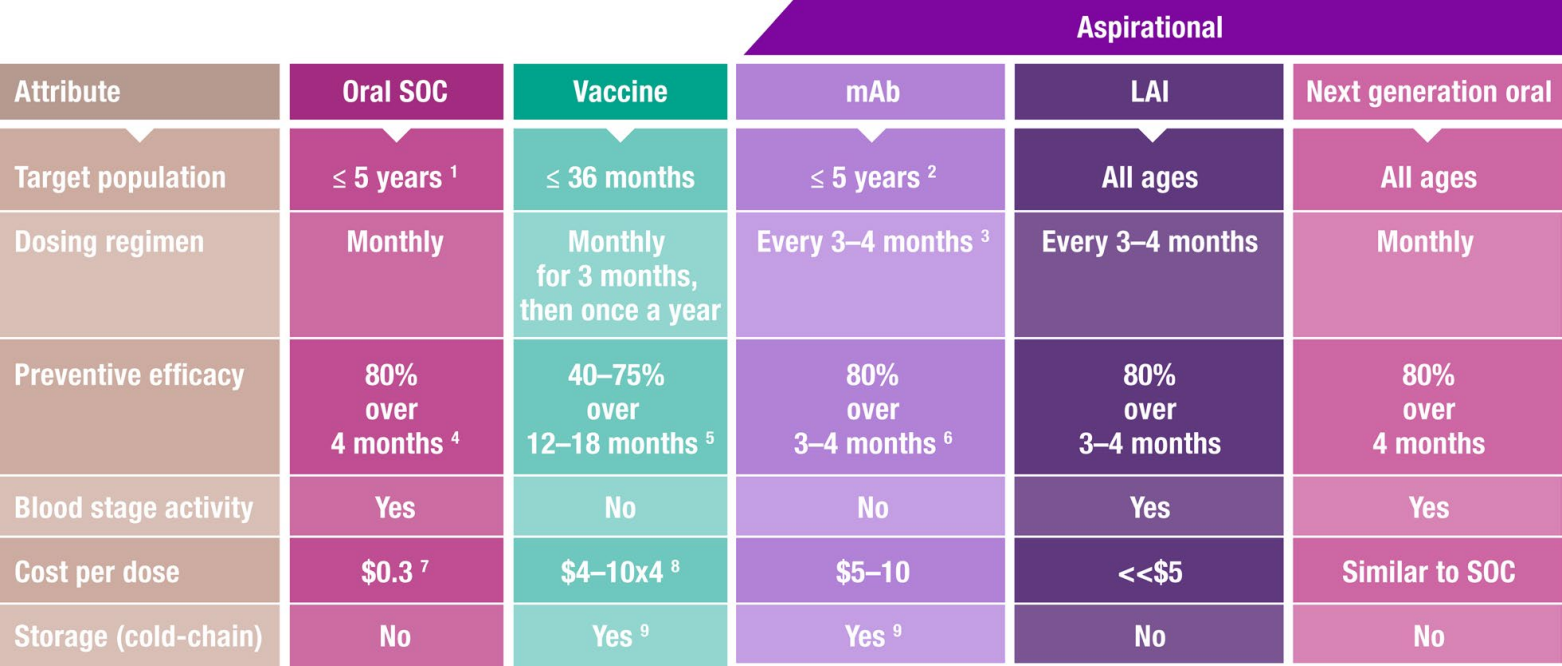
Main attributes of existing and future interventions for malaria prevention [63–68]. *SOC* standard-of-care, *mAb* monoclonal antibody, *LAI* long-acting injectable. ^1^Chemoprevention is recommended by WHO in children up to 15 years of age and pregnant women in their second and third trimester. SMC-SPAQ has been widely adopted, as compared to PMC and IPTsc, and was therefore considered as SOC. Main attributes for IPTp are similar and are not detailed in the summary Table. ^2^Most likely infants and children under 5 years of age in the first product label, due to injection volume and cost limitations. ^3^mAbs requiring more than one dose to protect throughout the malaria season may be considered based on cost-effectiveness [63]. ^4^Preventive efficacy of SPAQ was 83% in a controlled clinical trial [64]. ^5^RTS,S/AS01 preventive efficacy was of 39% for clinical malaria over 48 months [65]. R21/Matrix-M vaccine efficacy over 12 months was 75% at the seasonal sites and 68% at the standard sites for time to first clinical malaria episode [66]. ^6^WHO mAbs for malaria prevention preferred product characteristics, 2023 [63]. ^7^Average economic cost of dispersible SPAQ—26 cents per 3-days treatment course; and 28 cents for SP doses for adults [67]. ^8^RTS,S vaccine costs a maximum of EUR 9.30 per dose. R21 vaccine currently costs US$ 3.90 per dose for a two-dose presentation. Vaccines cost may decrease in future years as additional demand materializes [68]. ^9^Vaccines require a cold-chain for transportation and storage; and there are likely to be similar requirements for mAbs

The TPP is designed to set the expectations for the next generation of medicines. A previous TPP for oral chemoprevention was published by MMV in 2017 [60], and one for injectable chemoprevention including antibodies was published in 2018 [54]. These have formed the basis of discussions on preferred product characteristics (PPC) for chemoprevention convened by the WHO and MMV in 2020 [61, 62]. In the intervening period, the landscape has continued to evolve with the expansion of SMC, the approval by WHO of vaccines for infants, and the potential of mAbs and injectable long-acting medicines [2, 8, 57]. Additionally, data are available from market research conducted amongst healthcare providers from malaria endemic countries to better understand the preferred attributes for long-acting injectables (MMV, data on file). It is, therefore, timely to consolidate the outcome of various discussions on malaria prevention in a revised chemoprevention TPP. It is also important to consider to what extent these tools can be implemented in an integrated and complementary manner for maximal impact. A high-level summary (Fig. 2) compares the characteristics of the different interventions, underlining the potential of each approach.

A single TPP is proposed for long-acting oral and injectable drug combinations specifically developed for malaria chemoprevention both for populations living in endemic regions and for those from malaria-free areas. This latter group traditionally included travellers. However, as malaria elimination programmes progress, heterogeneity in transmission is inevitable. Thus, travellers within the same country moving from formerly endemic areas where malaria has been eliminated to regions still subject to ongoing transmission must be considered. With climate change, the frontier of malaria is moving upwards in altitude and latitude, exposing new populations to the malaria parasite for the first time [69]. Malaria epidemics associated with heavy rains and floods occurring in usually dry areas have also been reported [70].

Notably, all medicines currently used in chemoprevention were developed as treatments and repurposed for chemoprevention without stringent regulatory approval. Also, formerly published anti-malarial product profiles were conceptually built around atovaquone-proguanil malaria prophylaxis for travellers [54, 60]. Thus, the TPP is needed to support discussions around the development of potential regulatory pathways for new chemopreventive oral and long-acting injectable medicines.

### Updating the target product profile for malaria chemoprevention

#### Strategy for developing new malaria prevention tools

The simplest option for chemoprevention drug development is repurposing existing combinations approved for malaria treatment for use in chemoprevention [62]. However, this approach is sub-optimal, as medicines used for chemoprevention should ideally be different from those used for the treatment of malaria to mitigate against the risk of resistance selection [43].

The second approach is the recombine individual molecules from existing treatment combinations to make new products [62]. Although the safety of the individual medicines is well known, there is the potential for emergent adverse events with the new combination [71]. Thus, potential drug–drug interactions, safety, tolerability, and efficacy of the recombination must be evaluated. Again, there is a risk for resistance selection where chemoprevention drugs and treatment drugs contain the same active ingredients. From both a development and regulatory perspective, recombination is more demanding than repurposing [62].

The third strategy is the most challenging, but with greatest potential for developing products that address the limitations of current interventions. This requires identifying and developing new chemical entities and incorporating these into novel combinations specifically targeted at chemoprevention. This is a longer-term approach with a higher risk of attrition than repurposing or recombination, given the unknown clinical safety, tolerability and efficacy of the molecules [62]. It requires molecule optimization in the discovery phase to produce pharmacological duration of protection of at least a month. To facilitate this process, over the last five years, MMV has developed a modelling and simulation package, MMVSola, which enables the human dose to be estimated from metabolism and parasitology data (https://mmvsola.org/). This can be fine-tuned for molecules which have very low intrinsic clearance, the key parameter for long pharmacokinetic duration, and so will facilitate the optimization of new candidates for the pipeline.

Regardless of the drug development strategy used, or whether an oral or long-acting injectable formulation is developed, the revised TPP provides guidance to the desired attributes of the next-generation chemoprevention drugs (Table 1).

**Table 1:** Target product profile for malaria chemoprevention

Characteristics	Minimum essential	Ideal case
Indication for use	• Prevention against *P. falciparum* malaria	• Prevention against malaria caused by any *Plasmodium* species or mixed infection^a^
Target population	• Children ≥ 3 months to 10 years • OR pregnant women (second and third trimester) • OR non-immune individuals	• All age groups and populations at risk^b^ • Pregnant women in all trimesters and breastfeeding women
Dosing regimen	• Oral: once daily dosing for 3 consecutive days per month • Injectable: once every 3 months	• Oral: supervised single dose per month • Injectable: once every 6 months
Formulation	• Oral: co-packaged child-friendly formulation; tablets for adults • Injectable: intramuscular injection; volume injected and needle size in line with current standard for vaccines; partner drugs can be injected separately	• Oral: child-friendly fixed dose combination; tablets for adults • Injectable: liquid pre-filled injection device for intramuscular or subcutaneous injection; fixed dose combination of the drugs
Anti-malarial effects	• Two blood schizonticide drugs	• At least one drug with causal prophylactic, gametocytocidal or sporontocidal activities
Efficacy	• Clearance of existing asymptomatic infections • Paediatrics and non-immune: preventive efficacy against symptomatic malaria infections ≥ 90% over 1 month, or ≥ 80% over 4 months for orals; and ≥ 80% over 3–4 months for injectables • Pregnancy: preventive efficacy against symptomatic malaria infections in the mother of ≥ 75% over 6 months	• Paediatrics and non-immune: preventive efficacy against symptomatic malaria infections ≥ 75% over 6 months • Pregnancy: adverse pregnancy outcomes comparable to standard-of-care
Drug resistance	• Partner drugs with different modes of action • Active against known drug-resistant clinical isolates	• Partner drugs with similar pharmacological duration of efficacy^c^
Safety and tolerability	• Favourable risk–benefit profile, with safety and tolerability comparable to WHO recommended preventive treatment or standard-of-care	• Highly favourable risk–benefit profile with improved safety and tolerability versus WHO recommended preventive treatment or standard-of-care
Food effect (for oral medication)	• Easily implementable food recommendation	• No food recommendation
Drug–drug interactions	• Minimal interactions manageable with dose adjustments	• No clinically significant interactions
Cost of treatment course^d^	• Oral: $1 for adult, $0.25 for children ≤ 2 years • Injectable: equivalent or lower than malaria vaccine	• Oral: Lower than standard-of-care • Injectable: ≤ $1 for infants, ≤ $2 for children, ≤ $4 for adults
Stability^e^	• ≥ 2 years at ICH zone IVa/IVb conditions	• ≥ 3 years at ICH zone IVa/IVb conditions

^a^Activity against non-falciparum species would be demonstrated in vitro and would not be tested specifically in clinical studies

^b^High-risk groups, e.g., immunocompromised (HIV), sickle cell disease, malnourished

^c^Defined as time above the minimum inhibitory concentration

^d^Global Fund reference pricing for Q2 2024 (per treatment) is $0.28 SP doses for adults. For children, the cost for dispersible SP and dispersible SPAQ is $0.36 and $0.26, respectively. Gavi pricing of RTS,S/AS01 vaccine is EUR 9.30 per dose maximum. R21/Matrix-M vaccine currently costs US$ 3.90 per dose for a two-dose presentation, both prices being driven by the international market price for the adjuvants [68]. Note that pricing could be higher in premium private and traveller markets

^e^ICH zone IVa is hot/humid zone (30 °C and 65% relative humidity) and IVb is hot/higher humidity zone (30 °C and 75% relative humidity) defined according to the International Council for Harmonization of Technical Requirements for Pharmaceuticals for Human Use climate stability zone criteria

#### Target populations

The minimum essential target populations include those most at risk of malaria (Fig. 1). Different target populations will generate different risk:benefit requirements for drug development and one single product is unlikely to be suitable for all populations at risk. Thus, the TPP considers several options (OR statements) so that a drug which has value in one population is not discarded.

Children under 10 years of age living in malaria-endemic regions are particularly vulnerable to malaria infection and its associated complications. Malnourished children and those with sickle cell disease represent high-risk subgroups and should ideally be considered in ﻿product development. Recently WHO has included children at elevated risk of subsequent death from malaria due to underlying and unresolved anaemia as a special sub-group (post-discharge malaria chemoprevention).

Pregnant and breastfeeding women represent another important target population, especially women in their first trimester of pregnancy, who currently are left without any chemopreventive option. The Sustainable Development Goals promoting equity to maximize impact, underline the need to undertake research and fill the gap in this underserved population [22]. To address this need, compounds with no teratogenic signals in animal studies should be prioritized for development. Safety, tolerability, and efficacy data in the first trimester of pregnancy should be obtained as soon as possible. Typically, this requires sufficient data in non-pregnant women, women in the second and third trimester of pregnancy, and is ideally supported by inadvertent exposures from registries like the ongoing MiMBa pregnancy registry [22, 72]. In the initial stages of pregnancy, women are commonly reluctant to discuss or disclose their status or may not be aware of the pregnancy. Thus, all women of child-bearing age whose pregnancy status is unknown must ethically be treated as if they are potentially in the first trimester.

Beyond children and pregnant women, malaria chemoprevention would be valuable to any individuals without significant immune protection against the disease, including those living with HIV. Groups that could benefit from chemoprevention include migrant temporary workers, displaced people, nomadic populations, boarding school students, military personnel, and tourists travelling into malaria endemic regions. It also includes those who are impacted by climate change causing previously malaria-free altitudes and latitudes to become receptive to malaria transmission. As malaria elimination progresses, there will be an increasing number of older children for whom effective chemoprevention or vaccination has delayed the acquisition of semi-immune status, and adults where immunity to malaria has dissipated with declining transmission, or where there is a risk of re-establishment of transmission in regions where malaria has been locally interrupted.

#### Anti-malarial effects and efficacy

The primary parasite target population is *P. falciparum,* which causes almost 98% of the malaria cases, and most deaths*.* Thus, efficacy against this parasite, including against resistant parasites to currently available anti-malarial medicines, is the minimum requirement. However, in contrast to vaccines and candidate mAbs, new chemical entities are often effective against all *Plasmodium* species, and this should be considered a reasonable goal. *Plasmodium vivax* has become the most prevalent parasite in areas where malaria transmission is declining [73]. The same consideration applies to *Plasmodium ovale* and *Plasmodium malariae*, which are particularly relevant in the context of malaria elimination and often occur as co-infections with other *Plasmodium* species [74]. Routine surveillance of these parasites is limited in endemic regions, but *P. ovale* is the second most frequent cause of malaria in returning travellers to the UK [75]. There also remains the threat of primate malaria, such as *Plasmodium knowlesi,* whose long-term impact on malaria elimination remains unclear.

The ability to target multiple lifecycle stages is an advantage but not an absolute requirement. Chemoprevention originally relied purely on activity against blood stage parasites both for quinine family (quinine, chloroquine, amodiaquine and mefloquine) as well as the later sulfadoxine-pyrimethamine. Atovaquone-proguanil brought the additional benefit of activity against hepatic schizonts. For this revised TPP, a minimal essential profile would be two compounds with activity against parasite blood stages. However, compounds with activity against liver schizonts or even those with transmission blocking would bring additional value to the product [3]. Prevention of transmission would be of additional benefit in elimination efforts, and the prevention of re-establishment of malaria in populations with declining immunity [3].

In terms of clinical efficacy, long-acting oral and injectable drugs or prodrugs should clear pre-existing asymptomatic infections and provide long-lasting protection over weeks or months in endemic areas. Preventive efficacy may vary with local transmission dynamics, for example in seasonal versus holoendemic regions and in areas of low versus high transmission. Given that malaria transmission within countries becomes increasingly heterogeneous as malaria control and elimination efforts are enhanced, achieving sufficient preventive efficacy across various regions will be important to maximize community acceptance.

The target efficacy and duration of response is benchmarked against existing medications. The preventive efficacy achieved with SPAQ over a season in a controlled clinical trial (i.e., 80%, [64]) is proposed as minimum essential for oral drugs, in line with the WHO PPC [61]. Monthly oral chemoprevention should provide preventive efficacy of ≥ 90% over 1 month [61], or ≥ 80% over at least 4 months against symptomatic malaria infections in children and non-immune individuals. One major issue with SPAQ is that amodiaquine must be administered for three days per month; and this may contribute to lower effectiveness in real-life settings. Ideally, the next generation therapy should only require single-day monthly dosing. The target efficacy for long-acting injectable medicines would be similar with a preventive efficacy of 80% against clinical disease over 3–4 months with a single administration and is aligned with the WHO PPC for injectable mAbs [63]. This goal was originally set on the efficacy targets for pre-exposure prophylaxis in HIV, where 2–3 months was targeted with cabotegravir injectable [55]. More recent developments have led to lenacapavir, the first long-acting injectable HIV treatment medication administered twice yearly [76]. Similarly, a single injection that offers an efficacy of 75% over 6 months has been adopted as the ideal case for malaria chemoprevention in the TPP.

In pregnancy, women may have acquired partial immunity over time through repeated exposures and may have asymptomatic infection [77, 78]. This endangers the health and well-being of the mother, the fetus and the newborn child [79–81]. Therefore, oral or injectable drugs should provide at minimum a preventive efficacy of ≥ 75% against maternal malaria infections, regardless of symptoms, over 6 months. The incidence of placental malaria infection, severe malaria, hospital admission, death, and adverse pregnancy outcomes should also be considered as potential preventive outcomes in clinical trials [82]. From a public health perspective, chemoprevention in pregnancy should also improve maternal anaemia and birth outcomes, such as low birthweight. A reduction in the risk of low birthweight has been observed with the current standard-of-care SP, which may be mediated by its antibacterial efficacy [83]. However, it is important to underline that any regimen which can confer a malaria-free pregnancy for the mother is a significant breakthrough. Therefore, maternal malaria is recommended here as the primary endpoint for minimal acceptable chemopreventive efficacy.

#### Potential for drug resistance

Owing to the high number of individuals dosed, deployment of new chemoprevention drugs will inevitably reach individuals with high parasitaemia levels, increasing the risk of de novo resistance emergence. It is, therefore, fundamental that for any new compounds developed, there should ideally be no evidence of resistance selection in vitro or in vivo [84]. The minimal acceptable resistance threshold should have a value for the minimum inoculation for resistance (MIR) of 10^7^ and ideally undetectable resistant parasites recrudescing after clearance of 10^9^ parasites in culture (MIR > 9). Ideally, there should be no emergence of resistant parasites in monotherapy studies in mice, human volunteers, or patients, and there should be confirmation of activity against known drug-resistant clinical isolates.

Combination therapy will reduce the risk of infection by using two or more drugs with different modes of action and with different parasite resistance mechanisms. It is important to demonstrate that the second molecule is more active against resistant parasites generated by the first molecule, either because of increased sensitivity or increased fitness cost. Ideally, partner drugs should have matched pharmacological duration of cover, or time above the minimum inhibitory concentration, to ensure that neither drug is exposed to parasites as a pharmacological monotherapy. Parasite densities during the liver stage infection are low, so ideally if at least one of the drugs in the combination displays causal prophylaxis, this would reduce the potential selection of resistance during blood-stage infection when parasite densities are higher [3]. Finally, the drugs used for malaria chemoprevention should preferably not be used for case management in the same regions. This would reduce drug selection pressure for each drug class with the aim of retaining anti-malarial efficacy for longer.

#### Safety and tolerability

The clinical safety and tolerability requirements and risk–benefit balance for chemoprevention are more demanding than for malaria case management. Preventive medication is administered to healthy or asymptomatic individuals who are not at immediate risk of poor outcome. The safety and tolerability thresholds should therefore be comparable to that of established standard-of-care as a minimum, providing a favourable risk–benefit profile with only mild, transient drug-related adverse events and rare and manageable drug-related severe adverse events.

In terms of ideal characteristics, there are opportunities to improve safety, tolerability, and acceptability over the standard-of-care. For example, developing medicines with the potential for use throughout all trimesters of pregnancy would be a significant advance. Pregnancy testing is not always accepted, particularly for young unmarried women, and may not be available to identify women in their first trimester. For IPTp, current SP dosing is a single administration of three large tablets with 525 mg active ingredient. The tolerability and acceptability of an oral fixed-dose combination therapy need to be considered for pregnant women who may find taking oral medication difficult, and studies of recombining either mefloquine with SP or azithromycin with chloroquine have failed partly because of poor tolerability [85]. Notably, a long-acting injectable medicine or mAb does not have the issues of vomiting, pill burden, unpalatable ingredients and food effect that can impact adherence and efficacy for oral medications [57].

#### Food effect and drug–drug interactions

Ideally, oral chemoprevention should have no requirements for food intake. Any regimen that does not allow food before drug administration is likely to be unrealistic operationally since a fasting requirement is almost impossible to control, especially in children and pregnant women. However, food restrictions after oral drug administration may be acceptable if they are limited in duration. Without a full clinical assessment of the risk–benefit, it is difficult to set a numerical boundary on the fasting duration and degree of food effect. In case management, the food restrictions for piperaquine and lumefantrine which showed three-fold effects when administered with high-fat meal are manageable but not ideal [86, 87]. A food effect study with a high-fat meal should be conducted for all orally administered drugs [88]. However, the effect of a local diet should ideally be considered as it may be more pertinent to the habits in malaria-endemic regions. For example, no significant impact on piperaquine exposure was observed with local low-fat meals in South-East Asia [89].

Ideally, there would be no clinically significant drug–drug interactions affecting the efficacy or safety of anti-malarial therapy or essential concomitant medications. The target population may be exposed to concomitant medications like antibiotics, HIV and TB drugs, and oral contraception. Therefore, manageable drug–drug interactions with dose adjustments should be expected as a minimum. These restrictions are particularly important for the long-acting injectable medicines. In HIV PrEP, one of the main reasons for the successful deployment of injectable cabotegravir is the relative lack of drug interactions compared to previous HIV therapies.

#### Dosing regimen, formulation and stability

For an oral formulation, a directly observed single monthly dose would be consistent with the current standard-of-care in IPTp and PMC (i.e., SP). However, for SMC, a maximum of three consecutive days dosing once a day with a directly observed administration on the first day of dosing is the current standard-of-care (i.e., SPAQ) and is defined as the minimal acceptable profile. In addition, a 3-day dosing regimen would also be acceptable in cases where no other options are available or recommended. Oral products should preferably be formulated as fixed dose combinations. However, co-packaged partner drug formulations may be acceptable if fixed dose combination is not feasible. Formulations need to have high acceptability by the target population. Palatability and convenience are key considerations for chemoprevention to avoid sub-optimal dosing. For example, dispersible tablets can be given more easily to children and should be taste-masked and/or sweetened. Similarly, the pill burden needs to be acceptable, for example, in pregnant women who may be nauseous.

For a long-acting injectable product, market research showed that a 6-month duration of protection was perceived as ideal, and 3 months was acceptable (MMV, data on file). In addition, injections on two separate sites was deemed acceptable. The injection volume and needle size were of less importance, if they remained within the currently accepted standards (injection volumes from 0.5 mL to 2 mL, needle size of 25 gauge to 27 gauge). This is in line with the characteristics of most vaccines including RTS,S/AS01 and R21/Matrix-M, which are delivered via 0.5 mL intramuscular injections with a 25-gauge needle for each dose. Pre-filled syringes may be preferable to vials for ease of administration, but they increase the cost of the product, and depending on the target populations, multiple strengths may have to be developed.

It should be highlighted that the route of administration has an influence on the delivery channel. Oral medication can be delivered in very rural areas through community health workers making it more accessible than injectables which are currently administered by health care professionals at health facilities or advance via an health posts strategy where health facilities are too far or harder to reach.

Stability is important as malaria drugs will be deployed in areas that are hot and humid (zones IVa and IVb, with temperatures at 30 °C and relative humidity up to 75%). A minimum shelf life of 2 years at these conditions is necessary for both oral and injectable products. Accelerated stability data at 40°C/75% relative humidity should be generated to support short-term excursions, as it is often the case in some of the target areas of implementation.

#### Logistical and cost considerations

With a malaria prevention toolkit including vector control, vaccines, chemoprevention drugs, and potentially mAbs, the cost–benefit balance of the different options depending on operational circumstances will need to be understood. The costs of oral medication are likely to be less than those of an injectable chemoprevention drug and should be no more than $1 for adults and $0.25 for infants < 2 years old, and ideally not more than the current cost of standard of care, although this is most likely unachievable with any new chemical entities at launch. However, it is expected that long-acting injectables would be significantly cheaper than vaccines when produced at scale, which are price-limited by the high cost of the specialized adjuvants used. A target of $4 for adults, $2 for children and no more than $1 for infants per injection appears achievable within the public sector (Fig. 2). The cost ofmAb remains a challenge; at $50/g production costs, the use cases are limited to infants and newborns who would need low doses. Operational costs with long-acting injectable small molecules would be lower than for vaccines and mAbs since it is assumed they would not require such extensive cold storage and transportation. Thus, chemoprevention complements vaccines and mAbs in settings and populations where access to these interventions may be unfeasible or not cost-effective.

#### Malaria chemoprevention pipeline

**Fig. 3:**
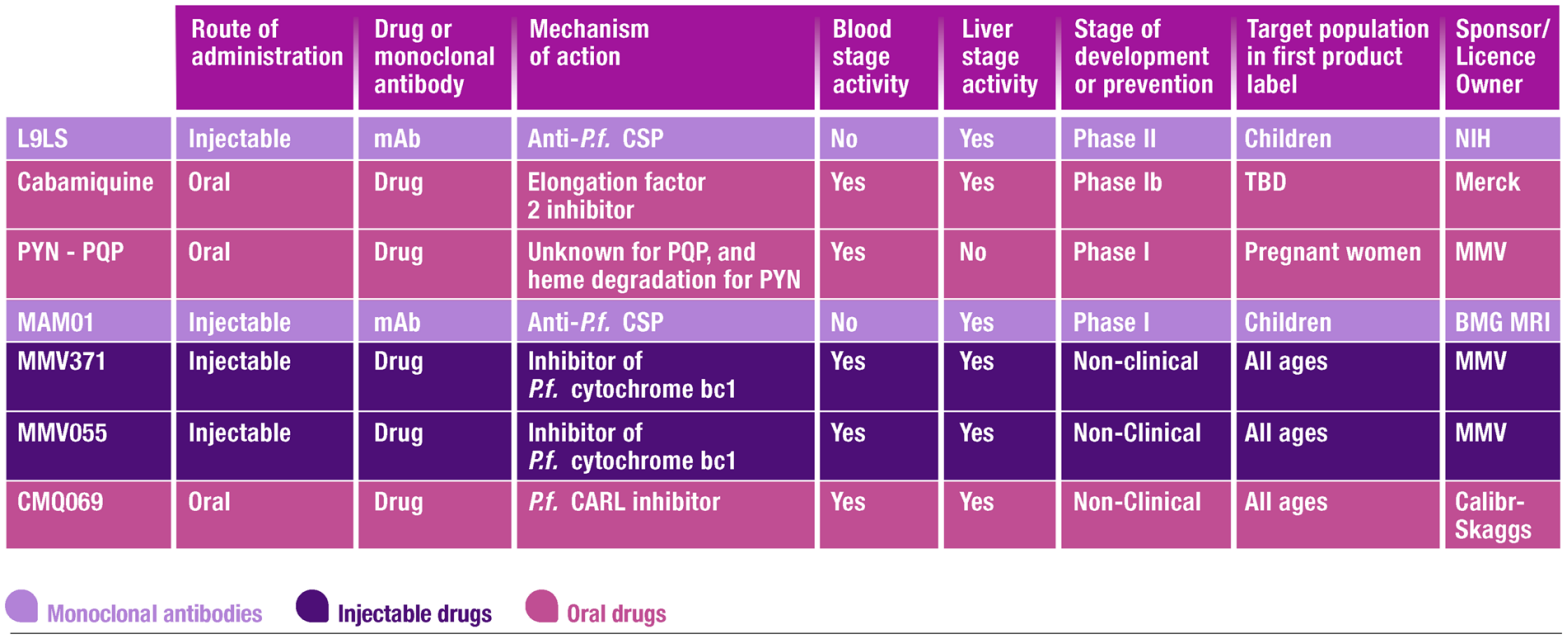
Research and development projects in malaria prevention (post-candidate selection). *BMG MRI* Bill & Melinda Gates Medical Research Institute, *CARL* cyclic amine resistance locus, *CSP* circumsporozoite protein, *mAb* monoclonal antibody, *MMV* MMV Medicines for Malaria Venture, *NIH* National Institutes of Health, *P.f.* *Plasmodium falciparum*, *PYN* pyronaridine, *PQP* piperaquine, *TBD* to be determined

The malaria prevention pipeline landscape is shown in Fig. 3.

Currently, the MMV chemoprevention portfolio for long-acting small molecule injectables includes MMV371 and MMV055 (also known as ELQ300) [90–93]. Both are potent inhibitors of *P. falciparum* cytochrome bc1, though with different binding site targets [94, 95]. MMV371, an ester-linked acetyl derivative of atovaquone, has completed preclinical safety testing and will progress into phase 1 first-in-human studies in 2024. Preclinical formulation development and testing of MMV055, both alone and in combination with MMV371, is ongoing.

For oral medicines, ganaplacide (KAF156) is a novel anti-malarial agent which belongs to the imidazolopiperazines class of anti-malarial drugs [96–98]. In a controlled human malaria infection (CHMI) model, KAF156 had an acceptable safety and tolerability profile and demonstrated high levels of pre- and post-CHMI protective efficacy [97]. Cabamiquine (M5717) is a first-in-class compound, targeting the *Plasmodium* cytosolic protein synthesis “Elongation Factor 2” for the treatment and chemoprevention of malaria [99–102]. A Phase 1b CHMI study showed that cabamiquine could be developed as a single-dose monthly regimen for malaria chemoprevention [102]. In addition, CMQ069, a *P. falciparum* cyclic amine resistance locus (*Pf*CARL) inhibitor, has recently successfully passed candidate selection. As *Pf*CARL confers parasite resistance to several anti-malarial drugs, inhibitors of this pathway could have utility in protecting partner drugs from resistance selection in combination [103, 104].

Molecules currently marketed or under development for case management may have potential for chemoprevention. A new combination of the existing anti-malarial actives pyronaridine and piperaquine was selected from all current recombining possibilities and is currently being investigated in phase 1 [105]. Both molecules have good tolerability, acceptable benefit:risk profiles, different mechanisms of action and similar long-lasting activity [86, 105–110]. Importantly, because neither molecule has shown any teratogenic liabilities [111, 112], or significant adverse events in pregnancy clinical trials and registries, the combination has potential for use in pregnant women in their first trimester of pregnancy.

Aside from small molecules, mAbs have been progressed into clinical trials for malaria prevention. The most advanced is L9LS, an anti-CSP-1 antibody which has shown high efficacy in volunteer infection studies and is now in two phase 2 studies in Africa (ClinicalTrials.gov Identifier: NCT05400655 and ClinicalTrials.gov Identifier: NCT05304611) [58, 113]. Further mAbs are in early stage of development, including MAM01/ATRC-501, an engineered version of a human antibody isolated from a RTS,S/AS01 vaccine trial participant, currently in phase 1 (ClinicalTrials.gov Identifier: NCT05891236) [114].

#### Considerations for drug development

The medicines currently used for chemoprevention are legacy medications, developed for the treatment of uncomplicated malaria and have not undergone stringent regulatory approval for chemoprevention. For example, SMC with SPAQ consists of a complete treatment course of amodiaquine plus SP given at monthly intervals. SP is used at its registered treatment dose and the dose of amodiaquine is the historical treatment dose. For future chemoprevention interventions, it may not be necessary to demonstrate efficacy for the treatment of acute uncomplicated malaria [62]. Instead, the clinical development strategy would aim for an indication of ‘prevention of malaria’, with a robust dosing justification, and will not have to be limited to a specific use case, such as SMC, PMC, IPTsc, or IPTp.

Developing drugs specifically for use in malaria prevention is a new concept. During scientific consultations with a stringent regulatory agency, no objections to the development of new chemical entities (NCEs) for malaria prevention without a treatment indication were indicated. In addition, utilizing the intramuscular route of administration for a long-acting NCE in a first-in-human study in the absence of prior oral clinical data was accepted provided that robust support from non-clinical data is available. Further discussions with stringent regulatory authorities, national regulatory bodies, and WHO will need to continue in concert with drug development to ensure a seamless and efficient progression. The regulatory path may be through a stringent regulatory authority, preferably by a process which includes WHO and participation of malaria-endemic country national regulatory authorities. For example, the European Medicines Agency EU-Medicines for all or 'EU-M4all' procedure [115], or the Swissmedic procedure for scientific advice and Marketing Authorization for Global Health Products [116], followed by WHO Prequalification [117]. However, the recent progress of the R21/Matrix-M vaccine demonstrates an alternative pathway, where the approval is granted by endemic countries ahead of stringent regulators, following their own analysis of the needs of their populations.

Clinical development of a novel anti-malarial will start with a phase 1 single ascending dose study in healthy adult participants to characterize the safety, tolerability and pharmacokinetics of the new molecules when administered alone and in combination. Physiologically-based pharmacokinetic (PBPK) modeling will be used to predict potential drug–drug interactions. The available non-clinical package and phase 1 clinical data will support a preliminary estimation of the preventive dose range and duration of protection before phase 2 programme initiation. Human volunteer infection studies may be conducted to further inform on the prophylactic activity and minimum efficacious concentration of the new agents. Exposure–response analyses of these studies may be used to support the dosing justification (doses and dosing regimen) and possibly to demonstrate the contribution of each individual agent to the preventive efficacy of the combination.

In malaria endemic regions, clinical trials will need to show preventive efficacy and evaluate the ability of the new combination to clear existing asymptomatic *P. falciparum* infections. The primary endpoint, comparator, malaria seasonality, transmission intensity, drug sensitivity patterns, expected level of efficacy and the size of the safety dataset will be key drivers for the sample size estimation to ensure adequate characterization of the safety and tolerability profile of the drug combination in the target population. The phase 2 and 3 clinical development pathway depends on the target population and the availability of standard-of-care. For example, in children, preventive efficacy is evaluated as an incidence rate ratio of symptomatic infections over 28 days and 4 months, as has been done for SMC with SPAQ [118]. In pregnant women, IPTp efficacy is primarily measured as maternal malaria incidence over the second and third trimester, i.e., a 6-month period [61]. Where no chemoprevention standard-of-care is implemented, placebo-controlled studies are preferred, enabling measurement of the baseline infection rate. The level of protection could then be established with the incidence rate ratio of clinical episodes and infections in the active treatment arm compared to the placebo arm. Where standard-of-care is available, non-inferiority to the recommended preventive treatment should be demonstrated and will inform the future decision on implementing the new product in the field.

Demonstrating safety in children and pregnant women presents challenges to drug development [22]. The size of the safety database adequately supporting the registration and/or recommendation of a specific chemoprevention combination will need to be discussed with regulators and policymakers. For compounds with clean teratogenic profiles in animal species, the generation of evidence in women in their first trimester of pregnancy should be initiated as soon as sufficient safety and efficacy data in non-pregnant adults and in pregnant women in second and third trimester are available [22]. Pregnancy registries collecting data from women inadvertently administered drugs during pregnancy could also contribute to risk assessment [72]. Prior to including women in the second or third trimester of pregnancy in a clinical trial, PBPK modelling will be used to predict if dose adjustments are necessary and to assess the potential foetal exposure to the tested drugs. As drugs may be given repeatedly, toxicities due to accumulation should be considered and ruled out in the pre-clinical setting/studies*.*

Efficacy, safety, and tolerability targets need to be considered in conjunction with key drivers of public health impact in the target population. For example, the convenience, acceptability, and feasibility of the dosing regimen and adherence when delivered through routine healthcare systems. It is also important to consider the presence of other preventive measures, the cost-effectiveness, coverage, and sustainability of the new intervention when delivered at scale, and operational implementation studies will be required.

## Conclusions

Highly effective preventive interventions have the potential to reduce the burden of malaria including severe cases and mortality. Current approaches have protected millions of people in malaria endemic countries, but the limitations of available interventions have resulted in inadequate deployment and restricted benefits for some key populations and settings. Also, the efficacy of current chemoprevention drugs is threatened by the emergence and spread of resistance. From the currently available technologies, or those which might be available in the future, long-acting chemoprevention drugs, either oral or injectable, by virtue of their simplicity and relative anticipated low cost, particularly compared to biologics, will be a critical element in the toolbox for malaria prevention. Even greater benefits are possible if such agents show improved efficacy, convenience, and feasibility over currently available drugs. As new tools emerge, it is essential to recognize that certain existing ones may become obsolete due to factors like drug resistance or limited acceptance. Innovation is occurring across all streams of malaria prevention, with new chemoprevention drugs, new vaccine development, and the opportunities for mAbs. The integration of these tools must be tailored to the target population and local conditions [119]. Ultimately, deploying and implementing malaria prevention interventions, drugs, vaccines, and mAbs in an integrated and complementary manner will fast track reduction of the disease burden, delay the onset of resistance to anti-malarial drugs and accelerate elimination of malaria in endemic countries.

## Data Availability

No datasets were generated or analysed during the current study.

## Abbreviations

CHMI: Controlled human malaria infection
HIV: Human immunodeficiency virus
IPTp: Intermittent treatment of malaria in pregnancy
IPTsc: Intermittent treatment of school aged children
MAb: Monoclonal antibody
MIR: Minimum inoculation for resistance
MMV: MMV Medicines for Malaria Venture
NCE: New chemical entity
PBPK: Physiologically-based pharmacokinetic
PPC: Preferred product characteristics
PMC: Perennial malaria chemoprevention
PrEP: Pre-exposure prophylaxis
SMC: Seasonal malaria chemoprevention
SP: Sulfadoxine-pyrimethamine
SPAQ: Sulfadoxine pyrimethamine plus amodiaquine
TPP: Target product profile
WHO: World Health Organization
