# Correction: Chemoprevention of malaria with long-acting oral and injectable drugs: an updated target product profile

**DOI:** 10.1186/s12936-024-05186-5

**Published:** 2024-11-29

**Authors:** Myriam El Gaaloul, Andre Marie Tchouatieu, Kassoum Kayentao, Brice Campo, Benedicte Buffet, Hanu Ramachandruni, Jean Louis Ndiaye, Timothy N. C. Wells, Celine Audibert, Jane Achan, Cristina Donini, Hellen C. Barsosio, Halidou Tinto

**Affiliations:** 1 MMV Medicines for Malaria Venture, Route de Pré‑Bois 20, Post Box 1826, 1215 Geneva 15, Switzerland; 2 Malaria Research and Training Center, University of Sciences, Techniques and Technologies of Bamako, Bamako, Mali; 3 University Iba Der Thiam of Thiès, Thiès, Senegal; 4 Malaria Consortium, London, UK; 5 Kenya Medical Research Institute, Centre for Global Health Research, Kisumu, Kenya; 6 Department of Clinical Sciences, Liverpool School of Tropical Medicine, Liverpool, UK; 7 Clinical Research Unit of Nanoro, Institut de Recherche en Sciences de la Santé, Nanoro, Burkina Faso

**Correction: Malaria Journal (2024) 23:315**10.1186/s12936-024-05128-1.

Following publication of the original article [1], it came to the authors’ attention that there was an error in Figure 3: in the ‘Liver stage activity’ column, the value ‘No’ was given for ‘MMV371’ and ‘MMV055’ rather than ‘Yes’, the correct value. The figure has since been updated. The authors thank you for reading this erratum and apologize for any inconvenience caused.

## References

[1] El Gaaloul M, Tchouatieu AM, Kayentao K, Campo B, Buffet B, Ramachandruni H, Ndiaye JL, Wells TNC, Audibert C, Achan J, Donini C, Barsosio HC, Tinto H. Chemoprevention of malaria with long-acting oral and injectable drugs: an updated target product profile. Malar J. 2024;23:315. 10.1186/s12936-024-05128-1. 39425110 10.1186/s12936-024-05128-1 PMC11490162

